# Antibiotic stewardship interventions in hospitals in low-and middle-income countries: a systematic review

**DOI:** 10.2471/BLT.17.203448

**Published:** 2018-02-28

**Authors:** Christophe Van Dijck, Erika Vlieghe, Janneke Arnoldine Cox

**Affiliations:** a Faculty of Medicine and Health Sciences, University of Antwerp, Wilrijkstraat 10, B-2650 Edegem, Belgium.; b Unit of Internal Medicine and Infectious Diseases, University Hospital Antwerp, Antwerp, Belgium.; c Unit of Tropical Laboratory Medicine, Institute of Tropical Medicine, Antwerp, Belgium.

## Abstract

**Objective:**

To review the effectiveness of antibiotic stewardship interventions in hospitals in low- and middle-income countries.

**Methods:**

We searched MEDLINE®, Embase®, Cochrane Central Register of Controlled Trials and regional indexes for studies of interventions to improve appropriate prescribing and use of antibiotics for hospitalized patients in low- and middle-income countries. We included controlled trials, controlled before-and-after studies and interrupted time-series studies published up to December 2017. We report prescribing, clinical and microbiological outcomes using a narrative approach.

**Findings:**

We screened 7342 original titles and abstracts, assessed 241 full-text articles and included 27 studies from 2 low-income and 11 middle-income countries. We found a medium (11 studies) or high risk (13 studies) of bias. Generally, all types of interventions (structural, persuasive and enabling) and intervention bundles were reported to improve prescribing and clinical outcomes. However, the studied interventions and reported outcomes varied widely. The most frequent intervention was procalcitonin-guided antibiotic treatment (8 of 27 studies, all randomized controlled trials). The intervention was associated with a relative risk for patients receiving antibiotics ranging between 0.40 and 0.87.

**Conclusion:**

The majority of studies reported a positive effect of hospital antibiotic stewardship interventions. However, we cannot draw general conclusions about the effectiveness of such interventions in low- and middle-income countries because of low study quality, heterogeneity of interventions and outcomes, and under-representation of certain settings. To strengthen the evidence base, action needs to be taken to address these shortcomings.

## Résumé

**Objectif:**

Étudier l'efficacité des interventions visant un usage plus rationnel des antibiotiques dans les hôpitaux de pays à revenu faible et intermédiaire.

**Méthodes:**

Nous avons consulté MEDLINE®, Embase®, le registre central Cochrane des essais contrôlés ainsi que des index régionaux afin de rechercher des études portant sur des interventions menées pour améliorer la prescription et l'usage des antibiotiques pour les patients hospitalisés, dans des pays à revenu faible et intermédiaire. Nous avons inclus des essais contrôlés, des études contrôlées avant/après et des études en séries temporelles interrompues, publiés jusqu'à décembre 2017. Nous évoquons ici, en adoptant une approche narrative, les résultats obtenus en termes de prescription et aux niveaux clinique et microbiologique.

**Résultats:**

Nous avons sélectionné 7342 résumés et titres originaux, évalué 241 articles dans leur version intégrale et inclus 27 études, pour 2 pays à faible revenu et 11 pays à revenu intermédiaire. Nous avons identifié un risque de biais moyen (11 études) ou élevé (13 études). En règle générale, ces publications indiquent que tous les types d'interventions (structurelles, persuasives et capacitantes) ainsi que toutes les interventions combinées ont permis d'améliorer les résultats en termes de prescription et au niveau clinique. Cependant, les interventions étudiées et les résultats publiés sont extrêmement variés. L'intervention la plus fréquente a consisté à guider les antibiothérapies en utilisant la procalcitonine (8 études sur 27; toutes correspondent à des essais contrôlés randomisés). Pour les patients, cette intervention a été associée à un risque relatif de prescription d’antibiotiques compris entre 0,40 et 0,87.

**Conclusion:**

La majorité des études font état d'un effet positif des interventions visant à promouvoir l'usage rationnel des antibiotiques en milieu hospitalier. Néanmoins, nous ne pouvons pas tirer de conclusions générales sur l'efficacité de ces interventions dans les pays à revenu faible ou intermédiaire, compte tenu de la mauvaise qualité des études, de l'hétérogénéité des interventions et des résultats ainsi que de la sous-représentation de certains contextes. Pour consolider les données disponibles, des actions doivent être entreprises afin de combler ces lacunes.

## Resumen

**Objetivo:**

Revisar la eficacia de las intervenciones de administración de antibióticos en hospitales de países con ingresos medios y bajos.

**Métodos:**

Se realizaron búsquedas en MEDLINE®, Embase®, en el Registro Central de Ensayos Controlados Cochrane y en índices regionales en relación a estudios de intervenciones para mejorar la prescripción y el uso adecuado de antibióticos para pacientes hospitalizados en países con ingresos medios y bajos. Incluimos ensayos controlados, estudios controlados de tipo antes y después y estudios de series de tiempo interrumpido publicados hasta diciembre de 2017. Informamos acerca de los resultados de prescripción, clínicos y microbiológicos usando un enfoque narrativo.

**Resultados:**

Revisamos 7342 títulos originales y resúmenes, evaluamos 241 artículos de texto completos, incluidos 27 estudios de 2 países con ingresos bajos y 11 con ingresos medios. Encontramos riesgo medio de sesgo (11 estudios) o riesgo alto (13 estudios). Por lo general, se informó de que todos los tipos de intervenciones (estructurales, persuasivas y permisivas) y conjuntos de intervenciones mejoran los resultados de prescripción y los resultados clínicos. Sin embargo, las intervenciones estudiadas y los resultados sobre los que se informó varían considerablemente. La intervención más frecuente fue el tratamiento antibiótico guiado de procalcitonina (8 de 27 estudios, todos ellos ensayos controlados aleatorizados). La intervención se asoció a un riesgo relativo para pacientes que recibían antibióticos que oscilan entre 0,40 y 0,87.

**Conclusión:**

La mayoría de los estudios informaron sobre un efecto positivo de las intervenciones hospitalarias con administración de antibióticos. Sin embargo, no podemos extraer conclusiones generales acerca de la efectividad de tales intervenciones en países con ingresos medios y bajos debido a la baja calidad del estudio, a la heterogeneidad de las intervenciones y los resultados y a la baja representación de ciertas regiones. Para fortalecer la base de las evidencias, es necesario tomar medidas para abordar estas deficiencias.

## الملخص

**الغرض:**

مراجعة فاعلية تدخلات الإشراف على المضادات الحيوية داخل المستشفيات في البلدان محدودة ومتوسطة الدخل.

**الطريقة:**

قمنا بإجراء بحث في MEDLINE®‎، وEmbase®‎، وسجل Cochrane المركزي الخاص بالتجارب المضبطة بالشواهد والمؤشرات الإقليمية الخاصة بدراسات التدخلات بهدف تحسين وصف الأدوية المناسبة واستخدام المضادات الحيوية للمرضى الذين تم إدخالهم المستشفيات في البلدان محدودة ومتوسطة الدخل. وقمنا بتضمين التجارب المضبطة بالشواهد، والدراسات القبلية والبعدية المضبطة بالشواهد ودراسات السلاسل الزمنية المتقطعة التي تم نشرها حتى شهر ديسمبر/كانون الأول 2017. ولقد أورد تقريرنا على محصلات مكروبيولوجية وسريرية ووصفية باستخدام النهج السردي.

**النتائج:**

كما قمنا بفحص 7342 من العناوين والملخصات الأصلية، وقمنا بتقييم 241 من المقالات النص الكامل، وتم تضمين 27 دراسة من اثنتين من البلدان محدودة الدخل و11 بلدًا من البلدان متوسطة الدخل. أظهرت (11 دراسة) مستوى متوسط من اختطار التحيز، أو (13 دراسة) مستوى مرتفع من اختطار التحيز. بشكل عام، تم الإبلاغ عن كل أشكال التدخلات (الهيكلية، والمقنعة، والتمكينية) ومجموعة التدخلات بهدف تحسين المحصلات السريرية والوصفية. ومع ذلك، فقد تباينت التدخلات والمحصلات المبلغ عنها بشكل كبير. وكان أكثر تدخل متكرر هو العلاج بالمضاد الحيوي الموجه بروكلسيتونين (8 من 27 دراسة، كل التجارب المعشاة المضبطة بشواهد). وارتبط التدخل بخطر نسبي على المرضى الذين يتناولون المضادات الحيوية تتراوح نسبته ما بين 0.40 و0.87.

**الاستنتاج:**

أبلغت معظم الدارسات عن وجود أثر إيجابي فيما يخص تدخلات الإشراف على المضادات الحيوية في المستشفيات. ومع ذلك، لا يمكننا الوصول إلى نتائج نهائية تخص فاعلية تلك التدخلات في البلدان محدودة ومتوسطة الدخل، بسبب الجودة النوعية المتدنية للدراسات، وعنصر عدم التجانس في التدخلات والمحصلات، والتمثيل غير الكافي لمناطق معينة. وحرصًا على تعزيز قاعدة الأدلة، فإننا نحتاج إلى اتحاذ الإجراءات اللازمة لتعويض هذا القصور. ****

## 摘要

**目的：:**

评价低收入和中等收入国家的医院对抗生素的管理干预措施的有效性。

**方法：:**

我们分别在 MEDLINE®、Embase®、Cochrane 临床对照试验中心注册数据库中进行检索，用于研究干预措施，以改善低收入和中等收入国家住院患者适当的开具处方和使用抗生素。我们包括了发表至 2017 年 12 月的对照试验、前后对照研究和断续时间序列研究。我们使用叙述性方法来报告处方、临床和微生物结果。

**结果:**

：我们筛选了 7342 条原标题和摘要，评估了 241 篇全文文献，其中包括来自 2 个低收入国家和 11 个中等收入国家的 27 项研究。我们发现了中等（11 项研究）或高等（13 项研究）偏倚风险。普遍来说，所有类型的干预措施（结构型、劝导型和授权型）和干预组合措施据报告可改善处方和临床结果。然而，研究的干预措施和报告的结果差异很大。最常见的干预措施是降钙素原指导的抗生素治疗（所有 27 例随机对照试验研究中有 8 例采用此干预措施）。此干预措施对接受抗生素治疗患者的相对危险范围为 0.40 至 0.87。

**结论：:**

大多数研究报告了医院抗生素管理干预措施的积极效果。然而，由于研究质量较低、干预措施及其结果的异质性以及某些背景的代表性不足，我们不能就这些干预措施在低收入和中等收入国家的有效性得出一般性结论。为了加强证据基础，需要采取行动来解决这些不足。****

## Резюме

**Цель:**

Провести анализ эффективности введения стратегии рационального использования антибактериальных препаратов в больницах в странах с низким и средним уровнем доходов населения.

**Методы:**

Авторы провели поиск в базах данных MEDLINE®, Embase®, Центральном Кокрановском реестре контролируемых испытаний и региональных предметных указателях на предмет исследований вмешательств с целью оптимизации назначения и использования антибиотиков для госпитализированных пациентов в странах с низким и средним уровнем доходов населения. Авторы включили контролируемые испытания, контролируемые исследования до и после вмешательства и исследования прерванных временных рядов, опубликованные до декабря 2017 года. Авторы сообщают о назначениях, результатах клинических и микробиологических исследований, используя описательный подход.

**Результаты:**

Авторы просмотрели 7342 оригинальных периодических издания и резюме, проверили 241 полнотекстовую статью и включили 27 исследований из 2 стран с низким уровнем доходов населения и 11 стран со средним уровнем. Был обнаружен средний (11 исследований) и высокий риск (13 исследований) систематической ошибки. В целом было отмечено, что все виды вмешательств (структурные вмешательства, убеждение и создание благоприятных условий), а также их комплексы оптимизировали назначение антибактериальных препаратов и клинические результаты. Тем не менее изученные вмешательства и зарегистрированные результаты сильно различались. Наиболее частым вмешательством было управление антибиотикотерапией на основе уровня прокальцитонина в крови (8 из 27 исследований, все — рандомизированные контролируемые исследования). Вмешательство было связано с относительным риском для получавших антибиотики пациентов, показатель которого находился в диапазоне от 0,40 до 0,87.

**Вывод:**

В большинстве исследований сообщалось о положительном эффекте введения стратегии рационального использования антибактериальных препаратов в больницах. Однако авторы не могут сделать общих выводов об эффективности таких вмешательств в странах с низким и средним уровнем доходов населения из-за низкого качества исследования, неоднородности вмешательств и результатов и недостаточного представления определенных параметров. Для укрепления доказательной базы необходимо принять меры для устранения этих недостатков.****

## Introduction

Antibiotic resistance is a problem of global importance.^1^ Representative data on the extent of the problem in low-and middle-income countries are relatively scarce, but high levels of resistance are increasingly being reported worldwide.^2^^–^^4^ Misuse and overuse of antibiotics in humans and animals is one of the main drivers of antibiotic resistance.^5^^,^^6^ Antibiotic stewardship, that is, interventions designed to optimize use of antibiotics, is therefore one of the key actions of the World Health Organization (WHO) Global Action Plan to contain antibiotic resistance.^5^^,^^7^ Stewardship interventions are typically classified as structural (such as the introduction of new diagnostic tests to guide antibiotic treatment), persuasive (such as expert audit of prescriptions and feedback advice to prescribers), enabling (such as guidelines or education on antibiotic use) or restrictive (such as expert approval for use of certain antibiotics).^8^ Often, different interventions are combined in antibiotic stewardship bundles.

Several systematic reviews showed that antibiotic stewardship interventions for hospitalized patients increased compliance with local antibiotic policies and improved clinical patient outcomes.^8^^–^^10^ These reviews included mainly or exclusively papers from high-income countries. Whether these results also apply to low- and middle-income countries is unclear. The organization of health-care system, availability of diagnostic testing and appropriate antibiotics, infection prevention and control practices and prescribing practices (such as over-the-counter availability of antibiotics) differs markedly between high-income countries and low- and middle-income countries.^11^ These differences may affect the implementation and effectiveness of antibiotic stewardship interventions in these settings.

Many hospitals in low- and middle-income countries are setting up antibiotic stewardship programmes.^12^ To better inform the selection of antibiotic stewardship interventions, we systematically reviewed the literature for studies that describe the effect of these interventions on clinical, microbiological or antibiotic prescribing outcomes in hospitalized patients in low- and middle-income countries.

## Methods

The review protocol including the complete search strategy has been registered at the PROSPERO international prospective register of systematic reviews (CRD42016042019).^13^

We included studies on antibiotic stewardship interventions for hospitalized patients in low- and middle-income countries. Stewardship interventions were defined as any intervention aiming to improve appropriate prescribing of antibiotics. A summary of the search strategy is shown in Box 1. Low- and middle-income countries were defined according to the World Bank criteria.^14^ To be included, studies had to report at least one prescribing outcome (such as defined daily doses per 100 bed-days), clinical outcome (such as mortality) or microbiological outcome (such as proportion of bacterial isolates with antibiotic resistance). We included (non)randomized controlled trials, cluster randomized controlled trials, controlled before‒after studies and interrupted time-series studies if these contained at least three points of comparison pre-and post-intervention. Studies were excluded if they included residents of long-term health-care or nursing facilities; studied malaria, human immunodeficiency virus, mycobacterial or fungal infections, *Helicobacter pylori* eradication, or care pathways (such as malnutrition bundles); compared antibiotic regimens; were written in language other than English, Dutch, French, German, Portuguese or Spanish; or had no full-text article available.

Box 1 **Search strategy for the review of antibiotic stewardship interventions in hospitals in low-and middle-income countries**

We searched the following databases from inception to 5 December 2017: Cochrane Central Register of Controlled Trials, EMBASE^®^, MEDLINE^®^, regional databases of the Global Index Medicus and the World Health Organization’s Virtual Health Library. The combination of the following and related terms was used: “low- and middle-income country”, “antibiotic”, “stewardship”, “inpatient” and terms related to study design such as “clinical trial”, “randomized controlled trial”, “interrupted time series”, “controlled before after”. Syntax and wording was adapted to the different libraries. Moreover, we searched reference lists of selected studies and of relevant reviews and consulted experts for additional literature. The full search strategy can be viewed online.^13^

Titles and abstracts were independently screened for eligibility by two authors. In case of disagreement, consensus was sought after reading the full-text article. The study selection was piloted by screening 630 abstracts and 44 full-text articles. These results were discussed among a panel of experts, after which the eligibility criteria were fine-tuned.

Two researchers extracted the data using an electronic form. The authors of original studies were not contacted in cases of incomplete or missing data. Data that were analysed inappropriately in the original studies were excluded. The quality of the studies was evaluated at the study level by two researchers independently. We used the 2017 quality criteria for randomized controlled trials and quasi-experimental studies of the Effective Practice and Organisation of Care Review Group.^15^ Reporting was done in line with the Preferred Reporting Items for Systematic Reviews and Meta-Analyses guidelines.^16^ For controlled trials, intention-to-treat analyses were reported unless indicated otherwise. If the original paper did not mention a relative risk (RR), we calculated a RR and 95% confidence interval (CI) if the necessary data were available. Due to the heterogeneity of the interventions and their reported outcomes, we present our findings using a narrative approach. Because of the large number of reported outcomes, we were unable to report all. We therefore selected the outcomes that were reported most frequently across the studies. We grouped studies by intervention type: structural, persuasive, enabling or intervention bundle.

## Results

**Fig. 1:**
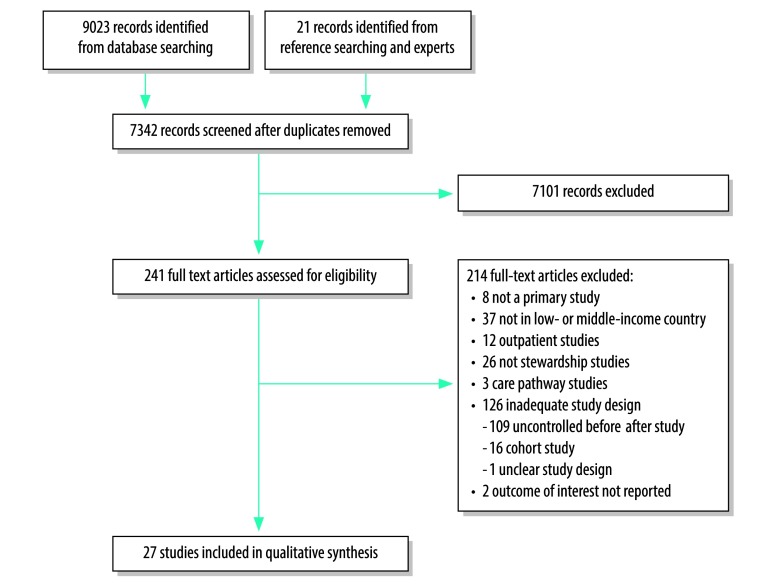
Flowchart of the selection of studies included in the review of antibiotic stewardship interventions in hospitals in low-and middle-income countries

We screened 7342 abstracts, selected 241 full-text articles and included 27 studies:^17^^–^^43^ 12 interrupted time-series, 9 randomized controlled trials, 3 cluster randomized controlled trials and 3 non-randomized controlled trials (Fig. 1). The studies were performed between 1996 and 2015 in 13 different countries. Two countries were considered low-income at the time of the study, one country transitioned from low to lower-middle income and the remaining were middle-income countries. Nine studies were conducted in multiple hospitals (range 2–65) but the majority was single-centre (18 studies). The interventions were implemented hospital-wide (10 studies) or on specific wards (17 studies) and targeted therapeutic prescriptions (20 studies), surgical prophylaxis (3 studies) or a combination of those (4 studies; Table 1).

**Table 1:** Characteristics of studies included in the review of antibiotic stewardship interventions in hospitals in low-and middle-income countries

Authors, year	Study design	Country	Setting	Participants	Intervention type	Intervention details	Target illness
Weinberg et al., 2001^39^	Interrupted time-series	Colombia	2 referral hospitals	Surgeons performing caesarean sections	Bundle	Guidelines on surgical antibiotic prophylaxis; structural changes (availability of prophylactic antibiotics on site); audit and feedback to physicians and nurses at hospital and individual level	Surgical site infections after caesarean section
Perez et al., 2003^40^	Interrupted time-series	Colombia	2 university hospitals	Hospital A: all prescribers; hospital B: anaesthesiologists	Bundle	Prescription form with (un)restricted antibiotics; educational campaign; reminders in the workplace	NR
Gülmezoglu et al., 2007^27^	Cluster randomized controlled trial	Mexico and Thailand	22 non-university maternity hospitals	Physicians, midwives, interns, students	Structural	Access to WHO’s online Reproductive Health Library^44^ and workshops on its use	Surgical site infections after caesarean section
Hadi et al., 2008^34^	Interrupted time-series	Indonesia	1 teaching hospital	All prescribers of 5 internal medicine wards	Enabling	Antibiotic guidelines; education for prescribers	NR
Özkaya et al., 2009^26^	Non-randomized controlled trial	Turkey	1 university hospital	Paediatric emergency department residents	Structural	Antibiotic initiation guided by influenza rapid test versus no laboratory investigation	Mild influenza-like illness
Rattanaumpawan et al., 2010^32^	Non-randomized controlled trial	Thailand	1 public university hospital	All prescribers	Persuasive	Audit and feedback to prescribers by infectious diseases specialist	NR
Long et al., 2011^18^	Randomized controlled trial	China	1 university hospital	Emergency department physicians	Structural	Antibiotic initiation and discontinuation guided by serum procalcitonin level versus routine care^a^	Community-acquired pneumonia
Maravić-Stojković et al., 2011^20^	Randomized controlled trial	Serbia	1 tertiary hospital	Cardiac surgery and intensive care unit staff	Structural	Antibiotic initiation guided by serum procalcitonin level versus routine care (based on clinical signs, C-reactive protein levels and leukocyte count)	Infections after coronary artery bypass grafting or valve surgery
Shen et al., 2011^33^	Cluster randomized controlled trial	China	1 tertiary hospital	All prescribers of 2 pulmonary wards	Persuasive	Audit and feedback to prescribers by clinical pharmacist	Respiratory tract infections
Opondo et al., 2011^37^	Cluster randomized controlled trial	Kenya	8 district hospitals	Nurses, clinical and medical officers	Bundle	Guidelines for treatment of non-bloody diarrhoea; education for prescribers; audit and feedback to prescribers on hospital performance	Non-bloody diarrhoea
Bucher et al., 2012^25^	Randomized controlled trial	Peru	1 public hospital	Paediatric emergency department physicians	Structural	Antibiotic initiation guided by faecal rotavirus rapid test in combination with a faecal leukocyte test versus faecal leukocyte test only	Acute diarrhoea
Magedanz et al., 2012^41^	Interrupted time-series	Brazil	1 university hospital	All prescribers of the cardiology department	Bundle	Restriction of certain antibiotics; audit and feedback to prescribers by (i) infectious diseases specialist and (ii) pharmacist	NR
Qu et al., 2012^24^	Randomized controlled trial	China	1 municipal hospital	Intensive care unit staff	Structural	Antibiotic initiation and discontinuation guided by serum procalcitonin level versus standard 14 days of antibiotics	Severe acute pancreatitis
Ding et al., 2013^17^	Randomized controlled trial	China	1 tertiary hospital	Respiratory ward physicians	Structural	Antibiotic initiation and discontinuation guided by serum procalcitonin level versus routine care (based on clinical experience, sputum bacteriology results and leukocyte count)	Acute exacerbation of idiopathic pulmonary fibrosis
Aiken et al., 2013^36^	Interrupted time-series	Kenya	1 public referral hospital	Nursing, medical and operating theatre staff	Bundle	Guidelines on surgical antibiotic prophylaxis; clinician education; patient education posters; audit and feedback to prescribers	Surgical site infections
Oliveira et al., 2013^23^	Randomized controlled trial	Brazil	2 public university hospitals	Intensive care unit staff	Structural	Antibiotic discontinuation guided by serum procalcitonin level versus C-reactive protein test	Sepsis or septic shock
Tang et al., 2013^21^	Randomized controlled trial	China	1 university hospital	Emergency department physicians	Structural	Antibiotic initiation guided by serum procalcitonin level versus routine carea	Acute asthma exacerbation
Chandy et al., 2014^35^	Interrupted time-series	India	1 private tertiary hospital	All prescribers	Enabling	Implementation and dissemination of antibiotic prescribing guidelines	NR
Long et al., 2014^19^	Randomized controlled trial	China	1 university hospital	Emergency department physicians	Structural	Antibiotic initiation guided by serum procalcitonin level versus routine care^a^	Acute asthma exacerbation
Najafi et al., 2015^22^	Randomized controlled trial	Islamic Republic of Iran	1 university hospital	Intensive care unit staff	Structural	Antibiotic initiation guided by serum procalcitonin level versus routine care^a^	Severe inflammatory response syndrome
Bao et al., 2015^42^	Interrupted time-series	China	65 public hospitals (30 tertiary; 35 secondary)	All prescribers	Bundle	Implementation of a nationally imposed multifaceted antibiotic stewardship programme	NR
Sun et al., 2015^43^	Interrupted time-series	China	15 public tertiary hospitals	All prescribers	Bundle	Implementation of a nationally imposed multifaceted antibiotic stewardship programme	NR
Gong et al., 2016^38^	Interrupted time-series	China	1 tertiary paediatric hospital	Paediatricians	Bundle	Antibiotic guidelines and prescribing restrictions; audit and feedback to prescribers by pharmacists and infection control physicians; financial penalties according to number of noncompliant prescriptions	NR
Brink et al., 2016^29^	Interrupted time-series	South Africa	47 private hospitals	Physicians, other clinical staff and managers	Persuasive	Audit and feedback to prescribers by a pharmacist	NR
Li et al., 2017^30^	Non-randomized controlled trial	China	6 university hospitals	Physicians of 8 intensive care units	Persuasive	Audit and feedback to prescribers by a pharmacist versus no intervention	NR
Tuon et al., 2017^28^	Interrupted time-series	Brazil	1 university hospital	All prescribers	Structural	Mobile phone application providing antibiotic prescribing guidance	NR
Wattal et al., 2017^31^	Interrupted time-series	India	1 tertiary hospital	Surgeons of 45 units	Persuasive	Audit and feedback to prescribers; focus group discussions per specialty	NR

NR: not reported; WHO: World Health Organization.

^a^ The content of routine care was not specified.

### Risk of bias assessment

**Fig. 2:**
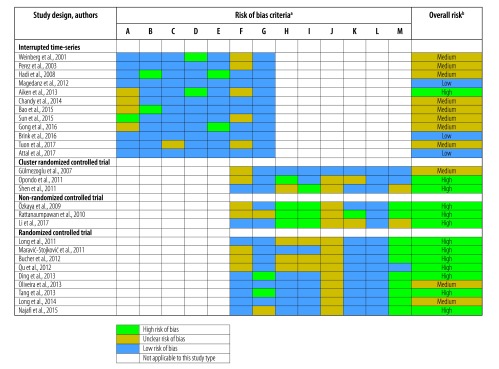
Assessment of risk of bias in studies included in the review of antibiotic stewardship interventions in hospitals in low-and middle-income countries

^a^ The criteria were: A: intervention independent of other changes; B: shape of intervention pre-specified; C: intervention unlikely to affect data collection; D: knowledge of allocated interventions adequately prevented during study; E: seasonality taken into account; F: incomplete outcome data adequately addressed; G: study free from selective outcome reporting; H: adequate allocation sequencing; I: adequate allocation concealment; J: baseline outcome measures similar; K: baseline characteristics similar; L: any blinding reported; M: study protected against contamination.

^b^ The risk of bias was considered low if all criteria were scored as low, medium if one or two criteria were scored as medium or high, and high if more than two criteria were scored as medium or high.^15^

For the 12 interrupted time-series studies the risk of bias was low (3 studies), medium (8 studies) or high (1 study; Fig. 2). The main risks of bias were that the intervention was not independent of other changes (5 studies) and that incomplete data were not adequately addressed (5 studies). For the 15 (non)randomized trials the risk of bias was medium (3 studies) or high (12 studies). The main risks of bias included the absence of baseline outcome measurement (14 studies), lack of protection against contamination (prescribers could have been involved in treatment of both the intervention and control group; 11 studies), non-random or unclear randomization methods (8 studies) and incomplete data not being adequately addressed (7 studies).

### Structural interventions

Structural interventions were reported by 12 studies,^17^^–^^28^ eight of which were randomized controlled trials of the effect of using serum procalcitonin levels to guide antibiotic treatment (Table 2).^17^^–^^24^ Five of these studies reported antibiotic use as the outcome. All of them found a significant decrease in the percentage of patients receiving antibiotics in the procalcitonin group compared with routine care or C-reactive protein testing. RR ranged between 0.40 and 0.87.^17^^–^^21^ Five studies reported patient deaths as the outcome and found no significant effect of procalcitonin-guided antibiotic use on in-hospital or 30-day mortality.^17^^,^^20^^,^^22^^–^^24^

**Table 2:** Outcomes of interventions to improve appropriate prescribing and use of antibiotics in hospitals in low-and middle-income countries: controlled trials

Intervention type and study design	Study duration, weeks	No. of patients	Data summary	Outcome measure	Effect size	*P*
**Structural intervention**						
Procalcitonin guidance						
Randomized controlled trial^18^	201	172	No. of patients receiving antibiotics: 72/86 in procalcitonin group; 79/86 in routine care group	RR of receiving antibiotic (95% CI)	0.87 (0.79 to 0.96)	0.01
Randomized controlled trial^20^	NR	205	No. of patients receiving antibiotics: 19/102 in procalcitonin group; 48/103 in routine care group	RR of receiving antibiotic (95% CI)	0.40 (0.25 to 0.63)	0.01
			No. of deaths: 3/102 in procalcitonin group; 3/103 in routine care group	RR of in-hospital death (95% CI)	0.88 (0.33 to 2.35)	0.80
Randomized controlled trial^17^	154	78	No. of patients receiving antibiotics: 26/39 in procalcitonin group; 35/39 in routine care group	RR of receiving antibiotic (95% CI)	0.74 (0.58 to 0.95)	0.01
			No. of deaths: 21/39 in procalcitonin group; 20/39 in routine care group	RR of death after 30 days^a^ (95% CI)	1.11 (0.76 to 1.64)	0.42
Randomized controlled trial^24^	133	71	No. of deaths: 7/35 in procalcitonin group; 8/36 in standard 14 days of antibiotics group	RR of in-hospital death (95% CI)	0.90 (0.37 to 2.22)	0.99
Randomized controlled trial^23^	141	97	No. of deaths: 21/50 in procalcitonin group; 21/47 in routine care group	RR of in-hospital death (95% CI)	0.92 (0.59 to 1.44)	0.84
Randomized controlled trial^21^	283	265	No. of patients receiving antibiotics: 59/132 in procalcitonin group; 95/133 in routine care group	RR of receiving antibiotic (95% CI)	0.63 (0.50 to 0.78)	0.01
Randomized controlled trial^19^	133	180	No. of patients receiving antibiotics: 44/90 in procalcitonin group; 79/90 in routine care group	RR of receiving antibiotic (95% CI)	0.56 (0.44 to 0.70)	0.01
Randomized controlled trial^22^	52	60	No. of deaths: 5/30 in procalcitonin group; 4/30 in routine care group	RR of in-hospital death (95% CI)	1.25 (0.37 to 4.21)	0.71
Rapid diagnostic testing						
Non-randomized controlled trial^26^	21	97	No. of patients receiving antibiotics: 34/50 in influenza rapid diagnostic test group; 47/47 in routine care group	RR of receiving antibiotic (95% CI)	0.68 (0.56 to 0.82)	0.01
Randomized controlled trial^25^	26	201	No. of patients receiving antibiotics: 29/100 in faecal leukocyte + rotavirus rapid test group; 50/101 in faecal leukocyte test only group	RR of receiving antibiotic (95% CI)	0.59 (0.41 to 0.84)	0.03
Library access plus workshops						
Cluster randomized controlled trial^27^	43 to 52^b^	1000 to 1022 per hospital	Mean % of operations with antibiotic prophylaxis: Mexico: 25.8 in intervention group; 6.5 in control group Thailand: 26.0 in intervention group; 14.7 in control group	% of operations with antibiotic prophylaxis: difference in adjusted rate (95% CI)	Mexico: 19 (−8 to 46)	0.12
					Thailand: 5 (−18 to 27)	0.66
**Persuasive intervention**						
Audit and feedback on individual patient cases						
Non-randomized controlled trial^32^	17	948	Mean no. of days of hospitalization: 30.4 in intervention group; 30.7 in control group	Mean difference in hospital length of stay (95% CI), days	−0.3 (−3.3 to −3.0)	0.80
			Mean no. of days of treatment: 12.7 in intervention group; 16.4 in control group	Mean difference in treatment duration, days	−3.7 (−5.2 to −2.2)	0.01
Cluster randomized controlled tria^33^	43	436	Mean no. of days of hospitalization: 14.2 in intervention group; 15.8 in control group	Mean difference in hospital length of stay (95% CI), days	−1.6 (−2.9 to −0.3)	0.03
Non-randomized controlled trial^30^	9	874	Median no. of days of treatment: 4.0 in intervention group; 5.0 in control group	Difference in median no. of days of treatment	1.0	0.03
**Intervention bundle**						
Treatment guidelines plus education plus audit and feedback						
Cluster randomized controlled trial^37^	77	1160	No. of patients receiving antibiotics for inappropriate indication: 313/594 in intervention group; 437/566 in control group	Absolute risk reduction for receiving antibiotic for inappropriate indication (95% CI)	41 (−6 to 88)	0.08

CI: confidence interval; DDD: defined daily doses; NR: not reported; RR: relative risk.

^a^ Per protocol analysis.

^b^ Different collection periods in different hospitals.

Note: Intention-to-treat analysis results are reported unless indicated otherwise. When significant *P*-values were not specified, we assumed *P* < 0.05 as significant.

A non-randomized controlled trial among 97 patients in a Turkish emergency department studied the effect of introducing a rapid diagnostic test for influenza-like disease.^26^ A lower percentage of tested patients were prescribed antibiotics compared with patients given clinical examination only (RR: 0.68; 95% CI: 0.56 to 0.82). In a randomized controlled trial among 201 patients in a Peruvian emergency department, use of a rapid test for rotavirus was associated with fewer patients receiving antibiotics (RR: 0.59; 95% CI: 0.41 to 0.84).^25^

In a cluster-randomized controlled trial in Mexico and Thailand health-care staff were given access to the WHO’s online Reproductive Health Library and workshops on its use.^27^ Thereafter, it was left open to the 22 participating hospitals whether certain activities, including antibiotic stewardship, were implemented. After 10‒12 months, no significant difference was found in the proportion of caesarean sections in which antibiotic prophylaxis was given, when comparing the 22 intervention hospitals to the 18 control hospitals (difference in adjusted rate in Mexico was 19.0%; 95% CI: −8.0 to 46.0% and in Thailand was 4.6%; 95% CI: −17.7 to 26.9%).

One interrupted time-series study evaluated the implementation of an antibiotic treatment guide through a free-of-charge mobile application (Table 3). Twenty-four months after the intervention there were significant increases in the defined daily doses per 1000 bed-days of recommended antibiotics (amikacin and cefepime) and a significant decrease in non-recommended antibiotics (piperacillin; *P* = 0.02). Use of other non-recommended antibiotics (meropenem, ciprofloxacin and polymyxin) did not decrease significantly.^28^

**Table 3:** Outcomes of interventions to improve appropriate prescribing and use of antibiotics in hospitals in low-and middle-income countries: interrupted time-series studies

Intervention	Study segments (duration in weeks)	No. of data points per segment (no. of observations per data point)	Outcome measure	Effect size^a^	*P*
**Structural interventions**					
Mobile phone application^28^	S1: Pre-intervention (52)	12 (NR)	DDD per 1000 bed-days	Baseline trend NR	N/A
	S2: Post-intervention (52)	12 (NR)		Trend increased for amikacin^b^	0.02
				Trend increased for cefepime^b^	0.01
				Trend decreased for piperacillin^b^	0.02
				Trend decreased for meropenem^b^	0.44
				Trend decreased for polymyxin^b^	0.34
				Trend decreased for ciprofloxacin^b^	0.08
**Persuasive interventions**					
Audit and feedback on individual patient cases^29^	S1: Pre-intervention (70)	16 (NR)	DDD per 100 bed-days	Baseline level NR	N/A
				Baseline trend +0.064/month	0.62
	S2: Implementation (104)	24 (NR)		Level change NR	N/A
				Trend change −0.56/month	0.01
	S3: Post-intervention (86)	20 (NR)		Level change NR	N/A
				Trend change −0.20/month	0.05
Audit and feedback at department level^31^	S1: Pre-intervention (52)	12 (NR)	DDD per 100 bed-days	Baseline level: NR	N/A
				Baseline trend: increasing in 1/35 wards^b^	0.05
	S2: Post-intervention (13)	3 (NR)		Level decreased in 3/35 wards^b^	0.05
**Enabling interventions**					
Treatment guidelines^34^	S1: Pre-intervention (16)	9 (14)	DDD per 100 bed-days	Baseline level: NR	N/A
				Baseline trend: −1.0 per 14 days	0.53
	S2: Guideline development (14)	6 (14)		Level change: −31.9	0.03
				Trend change +2.1 per 14 days	0.52
	S3: Guideline declaration (8)	4 (26)		Level change: −29.2	0.11
				Trend change: −9.5 per 14 days	0.14
	S4: Teaching sessions (8)	4 (27)		Level change: +38.2	0.05
				Trend change: +10.0 per 14 days	0.21
	S5: Refresher course (8)	5 (15)		Level change: −2.4	0.88
				Trend change: −9.8 per 14 days	0.15
Treatment guidelines^35^	S1: Pre-intervention (86)	20 (NR)	DDD per 100 bed-days	Baseline level: 56.9	N/A
				Baseline trend: +0.95 per month	0.01
	S2: Guideline preparation and booklet dissemination (94)	22 (NR)		Level change: NR	N/A
				Trend change: +0.21 per month	0.03
	S3: No new intervention (104)	24 (NR)		Level change: NR	N/A
				Trend change: +0.31 per month	0.01
	S4: Guideline revision and booklet dissemination (104)	24 (NR)		Level change: NR	N/A
				Trend change: +0.05 per month	0.64
	S5: Guideline revision and booklet with electronic dissemination (86)	20 (NR)		Level change: NR	N/A
				Trend change: −0.37 per month	0.01
**Intervention bundles**					
Treatment guidelines plus structural changes^39^	*Hospital A*				
	S1: Pre-intervention (13)	3 (308)	% of operations with surgical site infection	Baseline level: 13.9	N/A
				Baseline trend: NR^c^	NR
	S2: Guideline introduction with structural changes (30)	7 (272)		Level change: −9.8	0.01
				Trend change: NR^c^	NR
	S3: Post-intervention (21)	5 (217)		Level change: NR^c^	NR
				Trend change: NR^c^	NR
	*Hospital A*				
	S1: Pre-intervention (13)	3 (308)	% of caesarean sections with administration of antibiotic prophylaxis	Baseline level: 47.5	N/A
				Baseline trend: NR^c^	NR
	S2: Guideline introduction with structural changes (30)	7 (272)		Level change: +31.6	0.01
				Trend change: NR^c^	NR
	S3: Post-intervention (21)	5 (217)		Level change: −4.9	0.01
				Trend change: NR^c^	NR
	*Hospital B:*				
	S1: Pre-intervention (13)	3 (396)	% of caesarean sections with administration of antibiotic prophylaxis	Baseline level: 5.1	N/A
				Baseline trend: NR^c^	NR
	S2: Guideline introduction with structural changes (39)	9 (1026)		Level change: NR^c^	NR
				Trend change: +5.4 per month	0.01
	S3: Post-intervention (52)	12 (709)		Level change: +7.1	0.05
				Trend change: −4.1	0.01
	*Hospital A*				
	S1: Pre-intervention (13)	3 (308)	% of caesarean sections with administration of antibiotic prophylaxis within 1 hour of delivery	Baseline level: 32.5	N/A
				Baseline trend: NR^c^	NR
	S2: Guideline introduction with structural changes (30)	7 (272)		Level change: 62.2	0.01
				Trend change: NR^c^	0.01
	S3: Post-intervention (21)	5 (217)		Level change: NR^c^	NR
				Trend change: NR^c^	NR
	*Hospital B*				
	S1: Pre-intervention (13)	3 (396)	% of caesarean sections with administration of antibiotic prophylaxis within 1 hour of delivery	Baseline level: 30.8	N/A
				Baseline trend: +18.4 per month	0.01
	S2: Guideline introduction with structural changes (39)	9 (1026)		Level change: NR^c^	NR
				Trend change: −18.7 per month	0.01
	S3: Post-intervention (52)	12 (709)		Level change: +15.2	NR
				Trend change: NR^c^	NR
Prescription form plus education plus reminders^40^	S1: Pre-intervention (103)	103 (NR)	% of operations with incorrect timing of antibiotic prophylaxis	Baseline level: NR	N/A
				Baseline trend: NR	N/A
	S2: Post-intervention (42)	42 (NR)		Level change: −20	0.01
				Trend change: NR	NR
Antibiotic restrictions plus audit and feedback^41^	S1: Pre-intervention (129)	30 (NR)	Antibiotic use, DDD per 100 bed-days	Baseline level: NR	N/A
				Baseline trend +1.2 per month	0.01
	S2: Antibiotic restrictions plus audit and feedback by infectious diseases specialist (94)	22 (NR)		Level change: −1.3	0.8
				Trend change: −2.7 per month	0.01
	S3: Antibiotic restrictions plus audit and feedback by pharmacist (86)	20 (NR)		Level change: +4.7	0.4
				Trend change: +1.2 per month	0.01
Treatment guidelines plus education plus audit and feedback^36^	*Timing study*				
	S1: Pre-intervention (26)	26 (NR)	% of operations with incorrect timing of antibiotic prophylaxis	Baseline level: 99%	N/A
				Baseline trend: NR	N/A
	S2: Post-intervention (40)	40 (NR)		Level decreased^b^	0.01
				Trend decreased^b^	0.01
	*Infection study*				
	S1: Pre-intervention (26)	6 (223)	% of operations with surgical site infection	Baseline level: NR	N/A
				Baseline trend: −0.5 per month	0.49
	S2: Post-intervention (39)	9 (223)		Level change: NR	0.05
				Trend change: −0.7 per month	0.03
Multifaceted antibiotic stewardship programme^42^	*Outcome A*				
	S1: Pre-intervention (52)	12 (NR)	% of patients receiving antibiotic	Baseline level: NR	N/A
				Baseline trend +0.3 per month	> 0.05
	S2: Implementation (52)	12 (NR)		Level change: −2.3	> 0.05
				Trend change: −2.3 per month	0.01
	S3: Post-intervention (104)	24 (NR)		Level change: −2.7	0.05
				Trend change: +1.9 per month	0.01
	*Outcome B*				
	S1: Pre-intervention (52)	12 (NR)	Antibiotic use, DDD per 100 bed-days	Baseline level: NR	N/A
				Baseline trend: −0.4 per month	0.2
	S2: Implementation (52)	12 (NR)		Level change: +2.8	> 0.05
				Trend change: −2.2 per month	0.01
	S3: Post-intervention (104)	24 (NR)		Level change: −7.1	0.01
				Trend change: +2.4 per month	0.01
Multifaceted antibiotic stewardship programme^43^	S1: Pre-intervention (334)	26 (58)	% of patients receiving antibiotic	Baseline level: 74.7	N/A
				Baseline trend: −0.3 per quarter	0.01
	S2: Post-intervention (78)	6 (750)		Level change: −7.3	0.04
				Trend change: −1.5 per quarter	0.07
Treatment guidelines plus antibiotic restrictions plus audit and feedback^38^	S1: Pre-intervention (17)	4 (375 985)	% of patients receiving antibiotic	Baseline level: 59.0	N/A
				Baseline trend: −3.0 per month	0.01
	S2: Guidelines and restrictions (21)	5 (424 702)		Level change: +3.0	0.2
				Trend change: −0.4 per month	0.6
	S3: Financially punished audit and feedback (60)	14 (446 727)		Level change: −9.0	0.01
				Trend change: +3.0 per month	0.01

DDD: defined daily doses; N/A: not applicable; NR: not reported; RR: relative risk; S: segment.

^a^ In interrupted times-series studies the linear curve which summarizes the outcome data in each study segment can be defined by its level (*y*-intercept) and trend (slope). Level change reflects the difference of the level of the current segment compared with the level of the previous segment. Trend change reflects the difference of the trend of the current segment compared to the trend of the previous segment.

^b^ The authors reported no values for level or trend changes.

^c^ The authors reported that there were no significant changes but with no values for levels or trend changes.

### Persuasive interventions

Four studies evaluated the effect of audit and feedback to prescribers on individual patient cases by pharmacists (3 studies) or infectious diseases specialists (1 study):^29^^,^^30^^,^^32^^,^^33^ A non-randomized controlled trial including 577 patients in eight intensive care units reported a decrease of duration of antibiotic treatment of −1.0 day (*P* = 0.03) (Table 2).^30^ Another non-randomized controlled trial of 948 patients in a public university hospital reported a decrease of duration on antibiotic treatment of −3.7 days (*P* < 0.01) and a decrease in mean length of hospital stay of −1.6 days (*P* = 0.03).^32^ A cluster randomized trial found no significant difference in mean length of hospital stay among 436 patients (0.3 days; *P* = 0.8).^33^ An interrupted time-series study in 47 private hospitals in South Africa found a decreasing trend of antibiotic use during the implementation phase of the intervention (−0.56 defined daily doses per 100 bed-days per month; *P* < 0.01; Table 3).^29^ The trend was sustained in the 20 months post-implementation (−0.20 defined daily doses per 100 bed-days per month; *P* < 0.05).

An interrupted time-series study evaluated the effect of audit and feedback at the departmental level in 35 surgical wards. Three months after the intervention a significant decrease in defined daily doses per 100 bed-days was reported in 3 out of 35 wards (immediate decreases of −66.5%, −46.1% and −26.4% respectively; *P*  <  0.05).^31^

### Enabling interventions

Two interrupted time-series studied the effect of enabling interventions on antibiotic prescribing (Table 3).^34^^,^^35^ A study in an Indonesian hospital subsequently studied the development of treatment guidelines which were officially presented, followed by education and then refresher education. The authors reported a significant decrease of −31.9 defined daily doses per 100 bed-days (*P*  =  0.03) after guideline development and a significant increase of +38.2 defined daily doses per 100 bed-days (*P*  <  0.05) after education. The net effect of the intervention remains unclear.^34^ Another study in an Indian hospital evaluated the effect of an antibiotic policy guideline which was first developed and introduced, then revised and made available as booklet and lastly revised and made available through the intranet. The authors initially reported a baseline rising trend in antibiotic use of +0.95 defined daily doses per 100 bed-days per month (*P*  <  0.01) which levelled off after the first two interventions and declined by −0.37 defined daily doses per 100 bed-days per month (*P*  <  0.01) after the last intervention.^35^

### Intervention bundles

Eight studies evaluated bundles combining different interventions.^36^^–^^43^ A cluster randomized controlled trial in eight Kenyan hospitals compared a bundle containing guidelines, education and face-to-face feedback to prescribers with a similar, but less intensive bundle (fewer hours of training, written feedback; Table 2).^37^ Comparing prescriptions for 594 children in intervention hospitals and 566 children in control hospitals showed that the intensive bundle was associated with a non-significant absolute risk reduction in inappropriate use of antibiotics for non-bloody diarrhoea of 41% (95% CI: −6 to 88%).

The other seven studies all used an interrupted time-series design (Table 3). One study in two Colombian hospitals implemented antibiotic prophylaxis guidelines for caesarean sections, immediate availability of antibiotics in the operating theatre and feedback to surgeons about surgical site infections.^39^ The study reported a significant increase in the percentage of caesarean section births in which prophylaxis was administered (immediate increase by +31.6% in hospital A; *P* <  0.01 and gradual increase by +5.4% per month in hospital B; *P* <  0.01), an increase in antibiotic administration within 1 hour of delivery (immediate increase by 62.2% in hospital A only; *P* <  0.01) and a significant decrease in the monthly rate of surgical site infections with 9.8% (*P* <  0.01) in hospital A. 

In another study in a Kenyan hospital, surgical antibiotic prophylaxis guidelines were implemented, combined with training, personal feedback to prescribers and patient information posters.^36^ The proportion of operations with incorrect timing of antibiotic prophylaxis significantly decreased (no values reported) and the percentage of surgical site infections decreased after the intervention by −0.7% per month (*P  =*  0.03).

Another Colombian study introduced an antibiotic prescription form containing a list of restricted antibiotics with information on dosing intervals and an educational campaign.^40^ The study found a decrease of 20% (*P*  <  0.01) in the proportion of operations with incorrect timing of surgical prophylaxis.

In a Chinese study, guidelines and antibiotic restrictions were introduced, followed by individual prescriber audit and feedback, with financial penalties and revocation of prescribing privileges in case of non-compliance.^38^ Before the intervention the proportion of patients on antibiotic treatment was decreasing significantly by −3% per month from a baseline level of 59% (*P*  =  0.01). After the first intervention, no significant changes were reported. After the second intervention, a sudden drop of −9% (*P*  =  0.01) was observed, followed by a steady increase of +3% per month (*P*  =  0.01) in the next 14 months. The net effect of the intervention bundle remains unclear. 

A study in a Brazilian cardiology hospital first introduced restriction of certain antibiotics with individual audit and feedback to prescribers by an infectious diseases specialist and subsequently more comprehensive audit and feedback by a pharmacist. Before the intervention, the total antibiotic consumption significantly increased during 30 months (+1.2 defined daily doses per 100 bed-days per month; *P*  <  0.01). This trend decreased after the first intervention (−2.7 per month; *P*  <  0.01) and increased after the second (+1.2 per month; *P*  <  0.01). The net effect of the intervention bundle remains unclear.^41^

Two Chinese studies looked at the implementation of a multifaceted national antibiotic stewardship programme, containing structural changes, antibiotic restriction, education, guidelines, and audit and feedback, in 65 and 15 secondary and tertiary public hospitals respectively.^42^^,^^43^ Participation was compulsory and financial punishment for hospitals and disciplinary actions for individual prescribers could be imposed. Both studies reported a significant decrease in antibiotic use after the intervention. One study reported a decreasing trend of −2.2 defined daily doses per 100 bed-days per month (*P*  <  0.01).^42^ The other study reported a decrease in the proportion of patients receiving antibiotics (−7.3%; *P*  =  0.04).^43^

### Discussion

In this systematic review the majority of the included studies reported a positive effect of antibiotic stewardship interventions for hospitalized patients. This is in line with previously published systematic reviews on stewardship interventions in hospitals, which did not focus specifically on low- and middle-income countries.^8^^–^^10^ However, we cannot make general recommendations to guide the selection of antibiotic stewardship interventions due to limitations of the included studies, including the low quality of methods, variations and shortcomings in outcome reporting, under-representation of certain settings, heterogeneity of the interventions and variations in implementation strategy.

When screening titles and abstracts, we found 153 articles that reported on stewardship activities in a hospital setting, but 126 of those were excluded because of the study design (mainly bias-prone uncontrolled before‒after studies). So, although antibiotic stewardship is taking place and is being studied in low- and middle-income countries, most studies fall short methodologically. The studies we did include were also generally of low quality. For those with a randomized study design, a major risk of bias was contamination, meaning that prescribers could be involved in treatment of both the intervention and control groups. Because it may not be feasible to randomize individual prescribers, wards or hospitals to overcome this bias, interrupted time-series design has been recommended as an alternative. In interrupted time-series, data are collected continuously, and trends and outcome levels are compared before and after the intervention. To minimize bias and confounding, interrupted-time-series should meet certain requirements: a minimum of 12 data points before and after intervention, 100 observations per data point and the use of analytic techniques or models.^45^ These requirements were seldom met by the included studies. Poor quality of methods is a consistent theme among reviews of antibiotic stewardship in countries of all income levels and this issue needs to be addressed to strengthen the evidence base.^8^^,^^9^^,^^46^

Many of the included studies focused on a quantitative reduction in antibiotic prescribing. However, stewardship is not merely concerned with a reduction in antibiotic use, but in finding the balance between the potency of antibiotics and their potentially hazardous effects. The goal is to improve patient outcomes, decrease antibiotic resistance and increase cost‒effectiveness of care. Therefore, it is recommended that clinical outcomes (including adverse events), microbiological and cost‒effectiveness outcomes are reported in all stewardship studies.^8^^,^^47^ Most of the studies included in this review failed to do so. There is an ongoing debate about which parameters should be reported to accurately reflect the above-mentioned outcomes.^48^^,^^49^ This generally leads to a wide variety of reported parameters, as we observed in our review. This lack of uniformity limits comparison and aggregation of data. Also, for low- and middle-income settings, the measurement of certain clinical or microbiological outcomes, for example infection with *Clostridium difficile*, may be challenging if not impossible. Defining feasible outcome measures that can be uniformly applied in low- and middle-income countries should be prioritized. In the meantime, parameters that are easy to assess, such as mortality or hospital length of stay, should be reported by every stewardship study.

The majority of studies were performed in tertiary care centres in urban areas in middle-income countries, which limits the generalizability of the results. Large differences exist in terms of resources, organization, prescription practices and financing between countries and between facilities within countries.^11^ The intervention most frequently studied in our review was the implementation of procalcitonin testing. Although this intervention showed promising results, it may not be feasible to implement in many health-care settings in low- and middle-income countries. In addition, good quality evidence from non-tertiary or rural hospitals in low-income countries is lacking. Studies focusing on these settings should therefore be prioritized.

The effectiveness of the interventions varied across the studies, even those that implemented similar interventions. This is likely due to differences in the intervention or the implementation strategy, which may have been adapted to fit local circumstances. A detailed description of the intervention and the implementation strategy is therefore mandatory to interpret the study findings. Stewardship interventions in hospitals usually aim to change individual prescriber’s behaviour. This behaviour is influenced by social norms, attitudes and beliefs.^50^ These are therefore important determinants of the effectiveness of the intervention and should be an integral part of studies of stewardship interventions. For this reason, collaboration with behavioural scientists has been recommended.^46^ None of the included studies reported behaviour determinants.

Our review has several limitations. We defined a broad search strategy, allowing different settings, participants, interventions and outcomes to be included. This strategy provides a good overview of what evidence is available, but limits the generalizability of the findings. Moreover, to ensure the validity of the results, studies had to fulfil high methodological standards to be included. This led to discarding numerous lower quality studies. Also, we did not include studies that only reported cost (effectiveness) as an outcome, as these require a different analysis model. Lastly, due to publication bias (not reporting negative results) and language restrictions we may have missed certain studies.

We conclude that, based on the currently available evidence, general recommendations regarding the effectiveness of antibiotic stewardship interventions in low- and middle-income countries cannot be made. As many hospitals in low- and middle-income countries are setting up antibiotic stewardship programmes, what should be the way forward? On the basis of our findings, we suggest the following actions should be prioritized to strengthen the evidence base: (i) provision of methodological and statistical support for commonly used, complex study designs such as interrupted-time-series; (ii) seeking consensus on relevant and feasible outcome measurements for low- and middle-income countries; (iii) performing methodologically solid studies in settings such as non-tertiary, rural and public hospitals in low-income countries; and (iv) accurate descriptions of interventions, implementation strategies and inclusion of behavioural aspects. While awaiting the effect of these actions, the current lack of evidence should not prevent health-care workers from engaging in stewardship. Evidence and examples both from high- and low-and middle-income countries can inspire and provide guidance in the meantime.^8^^–^^11^
